# Changes in serum biomarkers of inflammation in bovine besnoitiosis

**DOI:** 10.1186/s13071-021-04991-0

**Published:** 2021-09-22

**Authors:** David González-Barrio, Ana Huertas-López, Carlos Diezma-Díaz, Ignacio Ferre, José Joaquín Cerón, Luis Miguel Ortega-Mora, Gema Álvarez-García

**Affiliations:** 1 Animal Health Department, Faculty of Veterinary Sciences, SALUVET, Complutense University of Madrid, Ciudad Universitaria s/n, 28040 Madrid, Spain; 2 Interdisciplinary Laboratory of Clinical Analysis (Interlab-UMU), Regional Campus of International Excellence Mare Nostrum, Espinardo, 30100, University of Murcia, Murcia, Spain

**Keywords:** Bovine besnoitiosis, Serological biomarkers, Acute-phase response, Haptoglobin, Albumin, Paraoxonase-1, Adenosine deaminase, Acetylcholinesterase

## Abstract

**Background:**

Acute and chronic besnoitiosis in extensive natural-service herds can have relevant effects in the health of bulls and negative consequences in their productive performance. Recent progress has been made in order to elucidate the pathogenesis of this disease. In this context, the study of biomarkers of inflammation in serum would contribute to gaining knowledge about the physiopathology of bovine besnoitiosis. Serological biomarkers could help in early diagnosis and prognosis, as seropositive bulls may have mild or severe testicular lesions.

**Methods:**

Herein*,* we have investigated the diagnostic and/or prognostic value of a panel of serum (serological) biomarkers related to inflammation, including total protein, globulin and albumin, haptoglobin (Hp), adenosine deaminase (ADA) paraoxonase-1 (PON-1) and acetylcholinesterase (AChE) in naturally and experimentally *B. besnoiti*-infected males classified according to different clinical phases of the disease (acute, chronic and subclinical besnoitiosis).

**Results:**

Results showed a similar response pattern in these biomarkers for naturally and experimentally infected cattle, with a few relevant variations. Most significant changes occurred during the acute phase of infection, although significant changes in a few biomarkers were also observed during the chronic infection. Haptoglobin, albumin, PON-1 and ADA were identified as the biomarkers that showed changes of higher magnitude in the acute phase of the infection, whereas high total protein and globulin values were found in chronically infected cattle. We have described the changes of a panel of inflammatory biomarkers of acute and chronic bovine besnoitiosis.

**Conclusions:**

In summary, several biomarkers with promising diagnostic value have been identified. The biomarkers associated with acute infection are related to previously reported molecular biomarkers in testicular parenchyma of infected bulls and could help in the diagnosis of early infections and complement results from specific immunoglobulin M (IgM) detection.

**Graphical abstract:**

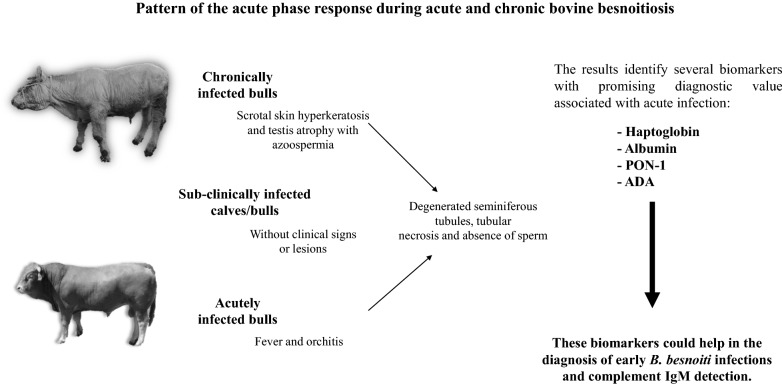

## Background

Bovine besnoitiosis caused by the protist cyst-forming coccidian parasite *Besnoitia besnoiti* is a chronic and debilitating disease. It is a well-known endemic cattle disease in sub-Saharan countries [1, 2] and has been spreading throughout different countries in Europe for the last two decades [3, 4] in the absence of vaccines and therapeutic tools. The disease progresses in two sequential steps. First, acute bovine besnoitiosis is characterised by fever, depression and anorexia followed by generalised oedema, ocular and nasal discharge, and orchitis [5, 6]. Next, chronic besnoitiosis is characterised by skin lesions such as hyperkeratosis, skin folding and alopecia that occur because of the development of tissue cysts in the connective tissues. Pathognomonic tissue cysts are visible in the ocular conjunctiva, mucous membranes of the upper respiratory tract and vestibulum vaginae. However, only a small proportion of animals show severe clinical signs and develop characteristic lesions, and most animals remain subclinically infected [7].

Bull infertility or even sterility with testicular degeneration and azoospermia is one of the most relevant economic consequences of *B. besnoiti* infection, particularly in extensive natural-service herds [8–11]. Bulls may already develop sterility during the acute phase as a consequence of vascular injury in the pampiniform plexus and scrotal skin lesions that hamper testicular thermoregulation [6]. In chronically infected bulls, numerous cysts are present in the scrotal skin, epididymis and ampullae and in the walls of blood vessels in the pampiniform plexus that may contribute to thermoregulation failure favoured by intense fibrosis and thickening of scrotal skin that interfere with normal spermatogenesis [8, 10, 12].

A relevant role of interstitial and recruited macrophages in the testicular parenchyma was recently suggested to contribute to the pathogenesis of infection at the testicular level, and different molecular markers were identified as prognostic indicators of sterility. In sterile bulls, the acute phase was mainly characterised by the upregulation of interleukin-1 alpha (IL-1α), IL-6 and matrix metalloproteinase inhibitor-1 (TIMP1), whereas in the chronic phase, the upregulation of intercellular adhesion molecules (ICAM) and the downregulation of metalloproteinase 13 (MMP13), tissue-type plasminogen activator (PLAT) and IL-1α were observed in the testicular parenchyma [13]. Since monitoring of disease progression in testicular parenchyma by measuring tissue markers may be difficult to implement as a routine diagnostic procedure, identification of markers in serum samples may be a more straightforward approach.

Acute or chronic systemic inflammation involves, among other events, an innate immune response through neutrophils or inflammatory cytokines produced by macrophages that enter the circulation and induce an oxidative response [14] and an increase in positive acute-phase proteins (APPs) and a decrease in negative APPs [15]. Since profiles involving various biomarkers rather than individual tests are recommended [15], we have investigated the diagnostic and/or prognostic value of a panel of biomarkers of inflammation in *B. besnoiti*-infected cattle grouped according to different clinical phases of the disease (acute, chronic and subclinical besnoitiosis). Haptoglobin (Hp), representative of positive APPs, albumin, representative of negative APPs, and paraoxonase-1 (PON-1), representative of oxidative stress markers, were measured. In addition, we determined the levels of globulins, total protein, and adenosine deaminase (ADA) and acetylcholinesterase (AChE), which are also related to the immune response and can be considered markers of inflammation [16].

## Methods

### Bovine serum panel and study design

Sera from experimentally (Table 1) and naturally (Tables 2 and 3) *B. besnoiti*-infected cattle were analysed. All our experimental procedures were approved by the Animal Welfare Committee of the Community of Madrid, Spain following procedures described in Spanish and EU legislation (PROEX 92/14, Law 32/2007, R.D. 53/2013), and Council Directive 2010/63/EU.

**Table 1:** Summary of the most relevant results regarding clinical signs, macroscopic and microscopic lesions, serological results and parasite detection in experimentally infected calves [17] and naturally infected bulls [6]

Group	No. animals	Systemic clinical signs/ lesions	Lesions in testicles		Parasite DNA detection		Immune response				
			Macroscopic lesions	Microscopic findings		Humoral				Cellular (IFN)	
						IgM	IgG	WB	IgG Avidity	Innate	Adaptive
G1 intravenous inoculation	3	Fever (1 day), lymphadenopathy, congestive ocular sclera, cough, nasal discharge and ocular tissue cysts	Not detected	Inflammatory infiltration, thrombus in epididymis and scrotum	Yes	Seroconversion at 12 dpi	Seroconversion at 19 dpi	Pos	Low	Peak at 12 dpi	Peak at 19 dpi
G2 subcutaneous inoculation	3	Fever (7 days), lymphadenopathy, congestive ocular sclera, Cough, nasal discharge ocular tissue cysts	Not detected	Inflammatory infiltration, oedema and thrombus in the scrotum	Yes	Seroconversion at 19 dpi	Seroconversion at 22 dpi	Pos	Low	Peak at 20 dpi	Peak at 12 dpi
G3 intradermal inoculation	3	Fever (8 days), lymphadenopathy, congestive ocular sclera, cough and nasal discharge, large number of ocular tissue cysts and skin lesions	Not detected	Inflammatory infiltration, oedema and thrombus in the scrotum and epididymis hyperkeratosis in scrotum and tissue cysts (average diameter of tissue cysts in G3 = 143.8 μm)	Yes	Seroconversion at 19 dpi	Seroconversion at 25 dpi	Pos	Low	Peak at 22 dpi	Peak at 19 dpi
G4 non-infected control group	3	No clinical signs and/or lesions	No lesions	No lesions	No	Seronegative	Seronegative	Neg	nd	Neg	Neg

*Pos* positive, *Neg* negative, *nd* not determined. More detailed data on experimental calf infection were previously published by Diezma-Diaz et al. [43]

**Table 2:** Summary of the most relevant results regarding clinical signs and serological results in acutely infected bulls [6, 13]

Group	Bull number	Systemic clinical signs/ lesions	Lesions in testicles		Parasite DNA detection	Immune response			
			Macroscopic lesions	Microscopic findings		Humoral			
						IgM	IgG	WB	IgG avidity
Early acute infection	A1^a^	Fever	Orchitis	nd	nd	Pos	Neg	Neg	nd
	A2	Fever	Orchitis	nd	nd	Pos	Neg	Neg	nd
	A3	Fever	Orchitis	nd	nd	Pos	Neg	Neg	nd
	A4	Fever	Orchitis	nd	nd	Pos	Neg	Neg	nd
	A5	Fever	Orchitis	nd	nd	Pos	Neg	Neg	nd
	A6	Fever, pneumonia, oedema in limbs	Orchitis, petechiae, haemorrhages and hydrocele	Vascular damage, inflammatory infiltrate, skin lesions and aspermia	Yes	Pos	Neg	Neg	nd
	A7	Fever	Orchitis	nd	nd	Pos	Neg	Neg	nd
	A8	Fever	Orchitis	nd	nd	Pos	Neg	Neg	nd
	A9	Fever, pneumonia, oedema in limbs	Orchitis, petechiae, haemorrhages and hydrocele	Vascular damage, inflammatory infiltrate and skin lesions	Yes	Pos	Neg	Neg	nd
	A10	Fever, pneumonia, oedema in limbs	Orchitis, petechiae, haemorrhages and hydrocele	Vascular damage, inflammatory infiltrate, skin lesions and aspermia	Yes	Pos	Neg	Neg	nd
	A11	Fever, pneumonia, oedema in limbs	Orchitis, petechiae, haemorrhages and hydrocele	Vascular damage, inflammatory infiltrate, skin lesions and aspermia	Yes	Pos	Neg	Neg	nd
Late acute infection	A1^b^	Fever	Orchitis	nd	nd	Pos	Pos	Pos	Low
	A2	Fever	Orchitis	nd	nd	Pos	Pos	Pos	Low
	A3	Fever	Orchitis	nd	nd	Pos	Pos	Pos	Low
	A4	Fever	Orchitis	nd	nd	Pos	Pos	Pos	Low
	A5	Fever	Orchitis	nd	Yes	Pos	Pos	Pos	Low
	A6	Fever, pneumonia, oedema in limbs	Orchitis, petechiae, haemorrhages and hydrocele	Vascular damage, inflammatory infiltrate, skin lesions and aspermia	Yes	Pos	Pos	Pos	Intermediate
	A12	Congestive scleral conjunctiva	Orchitis, hyperkeratosis, acanthosis and ulcers	Vascular damage, inflammatory infiltrate, skin lesions and tissue cysts (average diameter of tissue cysts = 56.6 μm)	Yes	Pos	Pos	Pos	Intermediate

*nd* not determined, *Pos* positive, *Neg* negative ^a^Bulls (A1–A6) were sampled twice at a 3-week interval as previously described in Diezma-Diaz et al. [44]. The second sampling was representative of a late acute infection, with all bulls showing IgG seroconversion

**Table 3:** Summary of the most relevant results regarding clinical signs and serological results in naturally infected bulls (chronically and subclinically infected bulls)

Group	No. animals	Systemic clinical signs/ lesions	Lesions in testicles		Parasite DNA detection	Serology			
			Macroscopic lesions	Microscopic findings		Humoral			
						IgM	IgG	WB	IgG avidity
Chronic infection	9	Folds and hyperkeratosis skin of perineum, carpus and tarsus, presence of cysts in scleral conjunctiva	Folds and hyperkeratosis in scrotum, skin of perineum, carpus and tarsus	Inflammatory infiltrate, skin lesions and tissue cysts (average diameter of tissue cysts = 191.0 μm)	Yes	Pos	Pos	Pos	High
Subclinical infection	34	No clinical signs or lesions	No lesions	Samples not available	nd	nd	Pos	Pos	nd^a^
Non-infected bulls	40	No clinical signs or lesions	No lesions	No lesions	nd	Neg	Neg	Neg	nd

*Pos* positive, *Neg* negative, *nd* not determined. ^a^Bulls with subclinical infection were IgG-seropositive in several consecutive samplings

#### Sera from experimentally infected calves

Sera were obtained from experimentally infected calves that developed a subclinical chronic infection [17]. Twelve healthy 3-month-old calves were randomly allocated into four different groups composed of three animals each (G1, G2, G3 and G4) (Table 1). The inoculum consisted of 10^6^ bradyzoites diluted in 2 ml of phosphate-buffered saline (PBS) administered through three different inoculation routes: intravenous inoculation by single jugular venepuncture (G1), subcutaneous inoculation in the left prescapular area (G2) and intradermal inoculation in the thigh area (G3). A non-infected control group was intravenously inoculated with 2 ml of PBS (G4). Daily clinical monitoring was carried out in all inoculated animals. Blood samples were collected on the day of inoculation, twice a week for the first month post-infection and once a week until the end of the assay at 70 days post-infection, with a total of 16 blood samplings for each animal. Five millilitres of peripheral blood in Vacutainer tubes (Becton, Dickinson and Company, Plymouth, UK) without anticoagulant was obtained by jugular venepuncture. Vacutainers without anticoagulant were centrifuged (1,200×*g* for 1 min) to obtain serum samples. A total of 64 serum samples were collected [17].

#### Sera from naturally infected animals

Animals were classified into different categories [bulls with (i) early acute infection, (ii) late acute infection, (iii) chronic infection, (iv) subclinical infection and (v) non-infected bulls] according to clinical signs and serological results. Molecular results as well as macroscopic and microscopic lesions were available and are shown in Table 2 for acutely infected bulls and Table 3 for chronically and subclinically infected bulls.

Twelve acutely infected bulls were analysed. All animals showed clinical signs characteristic of the acute phase, such as fever and orchitis. The acutely infected animals were divided into two subgroups as described in Table 2: (i) sera from 11 bulls that presented high levels of IgM without the detection of anti-*B. besnoiti* IgG (early acute infection [EA]) and (ii) sera from seven bulls with IgM and IgG levels (later acute infection [LA]).

Nine serum samples from seropositive chronically infected bulls were analysed. These animals showed clinical signs and lesions compatible with chronic besnoitiosis, such as scrotal skin hyperkeratosis and testis atrophy with azoospermia (Table 3).

Thirty-four serum samples from subclinically infected bulls were also analysed. These included seropositive fertile bulls without clinical signs or lesions (Table 3).

All animals came from extensive natural-service herds.

#### Sera from non-infected bulls

Forty serum samples from bulls that were seronegative for *B. besnoiti* and came from herds without a previous history of bovine besnoitiosis were included in this study (Table 3).

Blood samples were preserved at 4 °C until arrival at the laboratory and then centrifuged at 3000×*g* for 10 min, and serum was preserved at − 80 °C until further analysis.

The biomarkers measured were Hp, globulin, albumin, total protein, PON-1, ADA and AChE.

### Analytical methods

Analysis of the concentration of Hp, total proteins, albumin, globulin, serum PON-1 and AChE was performed in an automated analyser (Olympus AU 600, Beckman Coulter), following previously described methods [18–21]. In brief, the commercial Tridelta Phase range serum Hp kit (Tridelta Development Limited, Ireland) was used to determine Hp. This assay is based on the principle that the peroxidase activity of haemoglobin against inactivation is preserved when haemoglobin-Hp complexes are formed at low pH [18]. Total proteins and albumin were measured using commercially available reagents following the manufacturer’s instructions. The globulin concentration was calculated by subtracting the concentration of albumin from the total protein concentration. Serum PON-1 activity was determined by the analysis of the hydrolysis of *p*-nitrophenyl acetate to *p*-nitrophenol [19]. ADA activity was measured using an automated spectrophotometric method (Adenosine Deaminase Assay Kit, Diazyme Laboratories). This method determines the enzymatic deamination of adenosine to inosine, which after several reactions is transformed to *N*-ethyl-*N*-(2-hydroxy-3-sulfopropyl)-3-methylaniline and 4-aminoantipyrine. These molecules, in the presence of peroxidase, generate quinine dye, which is kinetically monitored at a 550-nm wavelength [20]. Finally, the AChE activity was analysed by measuring the hydrolyzation of butyrylthiocholine iodide (BTCI, Sigma) using 5,5′-dithiobis-2-nitrobenzoic acid (DTNB, Sigma) as chromophore [21].

### Statistical analysis

The levels of APPs (Hp and albumin), an oxidative stress marker (PON-1) and three additional markers of inflammation (globulin, AChE, ADA) were analysed by repeated-measures two-way analysis of variance (ANOVA) and Tukey’s post hoc tests in experimentally infected calves. The non-parametric Kruskal–Wallis test was used to compare naturally infected cattle groups, and Dunn’s post-test was used for multiple comparisons, as the data were not normally distributed. Two different analyses were performed in naturally infected bulls: (i) acutely (bulls A6, A10 and A11) and chronically infected bulls with testicular degeneration and subclinically fertile bulls were compared, and (ii) bulls classified in the different categories according to clinical signs and serological results [bulls with (i) early acute infection (*n* = 11), (ii) late acute infection (*n* = 7), (iii) chronic infection (*n* = 9), (iv) subclinical infection (*n* = 34) and (v) non-infected bulls (*n* = 40)] were included in the analyses. Statistical significance for the analysis was established with *P* < 0.05 using GraphPad Prism 6.01 software (San Diego, CA, USA).

## Results

Serological results showed a similar response pattern for naturally and experimentally infected cattle, with a few relevant variations. In both naturally and experimentally infected cattle, globulins, albumin and AChE showed significant variations. Haptoglobin was one of the most relevant markers in experimentally infected cattle, whereas ADA showed significant variations in naturally infected cattle. Most significant changes occurred during the acute phase of infection, although significant changes in a few biomarkers were also observed during chronic infection. The levels of each biomarker at 0 dpi in experimentally infected calves and in non-infected field cattle were very similar except for PON-1, which was lower in field animals than in experimentally infected calves.

### Acute-phase and oxidative stress responses in experimentally infected calves

**Fig. 1:**
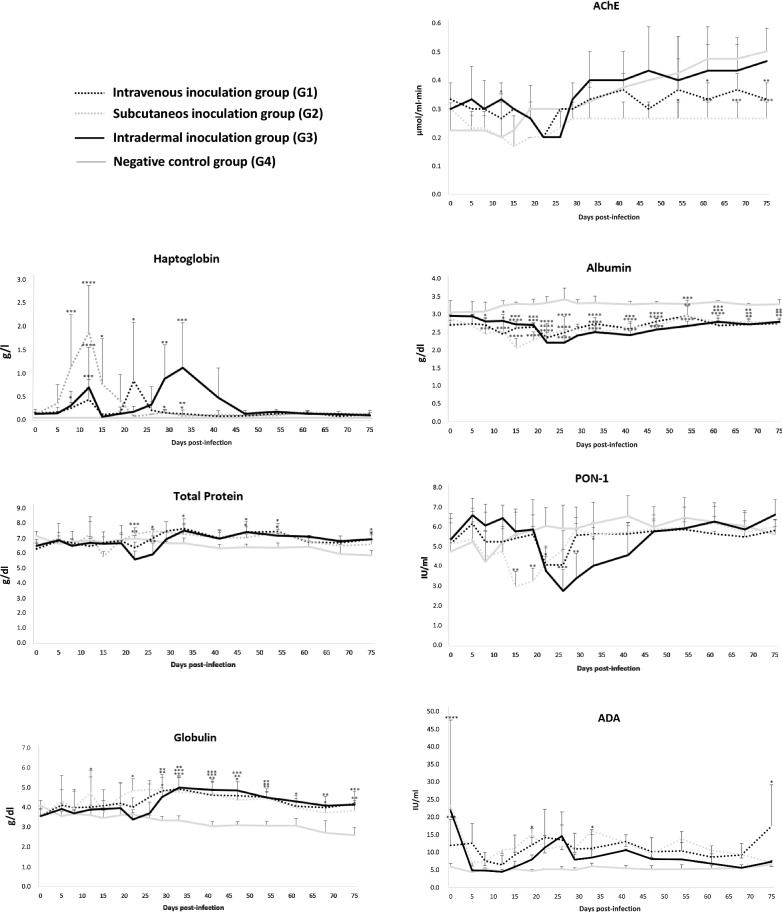
Serum concentrations of acute-phase response biomarkers in experimentally infected calves with 10^6^ bradyzoites inoculated by intravenous, subcutaneous and intradermal routes and in the control group. * *P* < 0.05, ** *P* < 0.01, *** *P* < 0.001, **** *P* < 0.0001

The serological results of the different markers are shown in Fig. 1.

Haptoglobin concentrations were increased from 8 to 12 days post-infection (dpi) in all infected groups. This increase was significant in the subcutaneously inoculated group at 8 dpi (ANOVA followed by Tukey’s post hoc multiple comparison tests, *q* = 5.88, *df* = 144, *P* < 0.001) and 12 dpi (ANOVA followed by Tukey’s post hoc multiple comparison tests, *q* = 10.02, *df* = 144, *P* < 0.0001), and concentrations decreased thereafter. In both the intravenously and intradermally inoculated groups, this increase was not significant at 8 dpi, and then the levels decreased, followed by a significant increase at 22 dpi in the intravenously inoculated group (ANOVA followed by Tukey’s post hoc multiple comparison tests, *q* = 4.318, *df* = 144, *P* < 0.05) and at 29 dpi (ANOVA followed by Tukey’s post hoc multiple comparison tests, *q* = 4.55, *df* = 144, *P* < 0.01) and 33 dpi (ANOVA followed by Tukey’s post hoc multiple comparison tests, *q* = 5.82, *df* = 144, *P* < 0.001) in the intradermally inoculated group. Next, Hp levels decreased until the end of the study.

Total protein levels were similar among the three inoculated groups with the exception of a few relevant findings when compared to the control group: higher levels at 54 dpi (ANOVA followed by Tukey’s post hoc multiple comparison tests, *q* = 3.83, *df* = 144, *P* < 0.05) in the subcutaneously inoculated group; higher levels at days 33, 47, 54 and 75 dpi (ANOVA, *F*_(15, 135)_ = 5.94, *P* < 0.05) in the intravenously inoculated group; and a significant decrease at 22 dpi (ANOVA followed by Tukey’s post hoc multiple comparison tests, *q* = 5.05, *df* = 144, *P* < 0.001), followed by an increase at 33 dpi (ANOVA followed by Tukey’s post hoc multiple comparison tests, *q* = 3.13, *df* = 144, *P* < 0.05) and maintenance of higher levels at 47 and 75 dpi (ANOVA, *F*_(15, 135)_ = 5.94, *P* < 0.05) in the intradermally inoculated group.

Globulin levels showed a significant increase compared to the control group from 22 dpi in the subcutaneously inoculated group (ANOVA, *F*_(15, 135)_ = 7.66, *P* < 0.05) and from 29 dpi in the intravenously (ANOVA, *F*_(15, 135)_ = 7.66, *P* < 0.01) and intradermally (ANOVA, *F*_(15, 135)_ = 7.66, *P* < 0.01) inoculated groups and thereafter.

AChE values increased from 25 dpi. In general terms, in all infected groups, the levels were similar to or lower than those detected in the control group. The most relevant differences corresponded to the subcutaneously and intravenously infected group at 47 dpi, showing lower levels than the control group (ANOVA, *F*_(15, 135)_ = 15.78, *P* < 0.05) until the end of the study, and in the intravenously infected group at 64 dpi (ANOVA, *F*_(15, 135)_ = 15.78, *P* < 0.01) and 75 dpi (ANOVA, *F*_(15, 135)_ = 15.78, *P* < 0.0001). The values increased similarly in both the intradermally infected group and the non-infected group.

The levels of albumin were lower in all inoculated groups than in the control group. This decrease was significant in the subcutaneously (ANOVA, *F*_(15, 135)_ = 6.53, *P* < 0.0001) and intravenously (ANOVA, *F*_(15, 135)_ = 6.53, *P* < 0.01) inoculated groups from 8 dpi onwards and in the intradermally inoculated group from 12 dpi onwards (ANOVA, *F*_(15, 135)_ = 6.53, *P* < 0.01).

Significantly lower PON-1 levels were observed at 15 (ANOVA, *F*_(15, 135)_ = 7.25, *P* < 0.01) and 19 dpi (ANOVA, *F*_(15, 135)_ = 7.25, *P* < 0.01) in the subcutaneously infected group and at 22, 26, 29 and 33 dpi (ANOVA, *F*_(15, 135)_ = 7.25, *P* < 0.05) in the intradermally infected group compared to the control group.

ADA levels were generally higher in all infected groups. The most relevant differences corresponded to 19 and 33 dpi in the subcutaneously infected group (ANOVA, *F*_(15, 135)_ = 3.33, *P* < 0.05) and to 75 dpi in the intradermally infected group (ANOVA, *F*_(15, 135)_ = 3.33, *P* < 0.05).

### Acute-phase and oxidative stress responses in naturally infected cattle

**Fig. 2:**
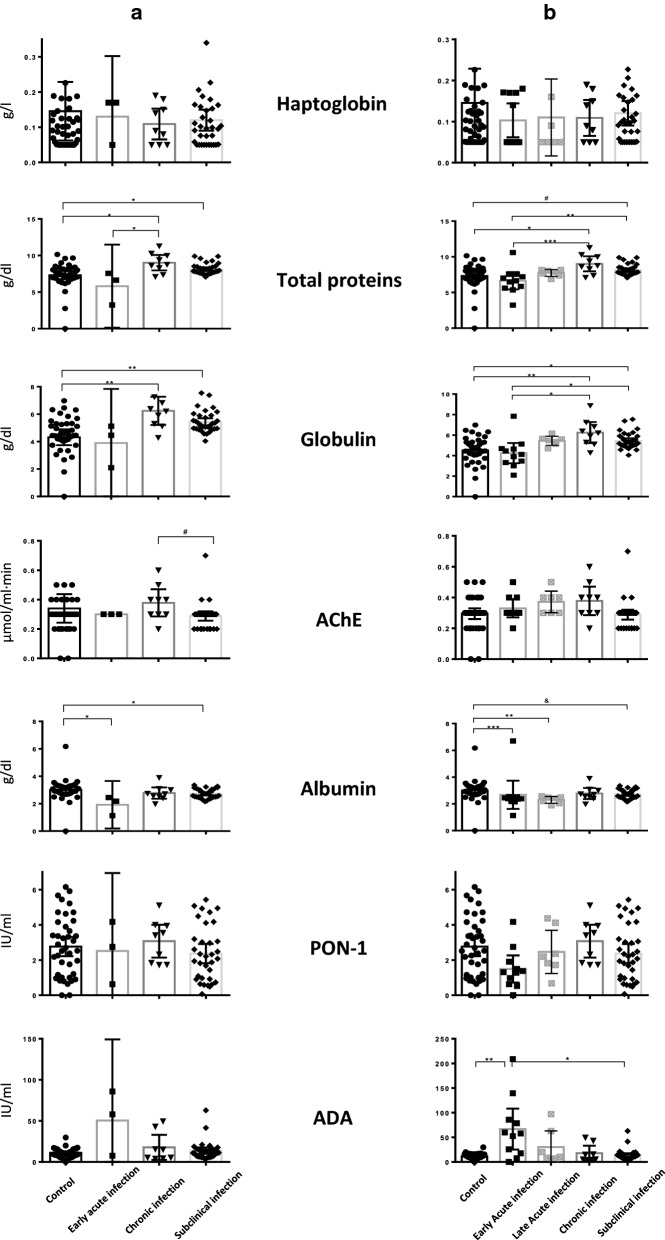
Serum concentrations of acute-phase response biomarkers in naturally infected and non-infected bulls. **a** Sterile bulls with acute or chronic infection and testicular degeneration and fertile subclinically infected bulls (seropositive without macroscopic lesions or clinical signs). **b** All bulls were classified according to clinical signs and serological results. Early acute infection: IgM+, IgG−, clinical signs compatible with an acute infection. Chronic infection: IgM+, IgG+, high IgG avidity index, clinical signs and lesions compatible with a chronic infection. Subclinical infection: IgM+, IgG+, high IgG avidity index, absence of clinical signs and lesions compatible with a chronic infection. ^#^*P* = 0.07; ^&^*P* = 0.06; *P < 0.05, **P < 0.01, ***P < 0.001, ****P < 0.0001

The serological results of the different markers are shown in Fig. 2. Similar results were obtained regardless of the criteria employed to classify the animals in the different categories (panel a: sterile acutely and chronically infected bulls versus fertile subclinically infected bulls; panel b: bulls with acute, chronic or subclinical infection based on clinical signs and serological results). However, differences were more evident in panel b when animals were classified according to serological results and clinical signs compared to a more restrictive criterion where only sterile and fertile bulls were considered.

Non-significant variations in Hp, AChE and PON-1 values were observed among the groups studied (Fig. 2). The remaining markers showed significant differences between some of the groups, as mentioned below.

Total protein levels were higher in chronically infected bulls than in non-infected bulls (Kruskal–Wallis *H*-test followed by Dunn’s multiple comparison test: *χ*^2^ = 28.66, *df* = 2, *P* < 0.05) and acutely infected bulls (Kruskal–Wallis *H*-test followed by Dunn’s multiple comparison test: *χ*^2^ = 15.51, *df* = 2, *P* < 0.05) as shown in Fig. 2a. These values were similar in those groups with clinical signs (Fig. 2b) in which levels of total protein were higher in chronically infected and subclinically infected bulls than in non-infected bulls (Kruskal–Wallis *H*-test followed by Dunn’s multiple comparison test: *χ*^2^ = 32.93, *df* = 2, *P* < 0.05 and *χ*^2^ = 18.37, *df* = 2, *P* < 0.07, respectively) and early acutely infected bulls (Kruskal–Wallis *H*-test followed by Dunn’s multiple comparison test: *χ*^2^ = 51.28, *df* = 2, *P* < 0.001 and *χ*^2^ = 36.72, *df* = 2, *P* < 0.01, respectively).

Globulins levels were significantly higher in chronically and subclinically infected bulls than in non-infected bulls (Kruskal–Wallis *H*-test followed by Dunn’s multiple comparison test: *χ*^2^ = 32.78, *df* = 2, *P* < 0.01 and *χ*^2^ = 19.26. *df* = 2, *P* < 0.01, respectively) (Fig. 2a). The similar is found in bulls with clinical signs (Fig. 2b), where significantly higher levels in were chronically and subclinically infected bulls than in non-infected bulls (Kruskal–Wallis *H*-test followed by Dunn’s multiple comparison test: *χ*^2^ = 38.15, *df* = 2, *P* < 0.01 and *χ*^2^ = 22.44, *df* = 2, *P* < 0.05, respectively) and early acutely infected bulls (Kruskal–Wallis *H*-test followed by Dunn’s multiple comparison test: *χ*^2^ = 48.12, *df* = 2, *P* < 0.01 and *χ*^2^ = 32.40, *df* = 2, *P* < 0.05, respectively).

Higher values of AChE but not significant were observed in late acute and chronic infections (Fig. 2).

Albumin values were lower in all infected groups than in the non-infected group. These differences were significant in acutely infected bulls [acute infection: Kruskal–Wallis *H*-test followed by Dunn’s multiple comparison test: *χ*^2^ = 45.53, *df* = 2, *P* < 0.05 (Fig. 2a); early acute infection: *χ*^2^ = 4.15, *df* = 2, *P* < 0.001 (Fig. 2b); late acute infection: *χ*^2^ = 45.43, *df* = 2, *P* < 0.01 (Fig. 2b)] and in subclinically infected bulls (*χ*^2^ = 17.17, *df* = 2, *P* < 0.05 in Fig. 2a, and *χ*^2^ = 18.79, *df* = 2, *P* < 0.06 in Fig. 2b) compared to non-infected bulls.

Non-significant variations in PON-1 values were observed among the groups studied (Fig. 2a, b). The highest levels corresponded to bulls with chronic infection, followed by bulls with acute infection and subclinically infected bulls (Fig. 2a). The lowest levels corresponded to bulls with early acute infection (Fig. 2b).

Finally, the highest ADA levels corresponded to bulls with an early acute infection, followed by bulls with a late acute infection, chronically infected bulls and finally subclinically infected cattle. Significant differences were observed between bulls with an early acute infection and subclinically infected bulls (Kruskal–Wallis *H*-test followed by Dunn’s multiple comparison test: *χ*^2^ = 29.55, *df* = 2, *P* < 0.05) and the negative control (*χ*^2^ = 33.66, *df* = 2, *P* < 0.01).

## Discussion

We have studied for the first time a panel of serum biomarkers representative of either APPs or oxidative stress response in the different clinical scenarios of bovine besnoitiosis (acute, chronic and subclinical infection). In the present study, several biomarkers showed significant variations in infected animals with a slightly different profile for naturally and experimentally infected cattle.

The search for serum biomarkers can help with early diagnosis and elucidate the clinical and molecular pathogenesis puzzle of bovine besnoitiosis [6, 22]. Previous attempts have focused on haematological and biochemical parameters [23, 24]. However, changes in a few biomarkers were observed when animals with well-defined acute, subacute and chronic besnoitiosis were studied compared to another study where only seropositive cattle were compared with seronegative cattle [23, 24]. Therefore, a well-characterised panel of clinically affected animals is more convenient in the search for biomarkers, and acute-phase response markers could be of value in bovine besnoitiosis, where a systemic inflammatory reaction is especially intense during the acute phase and focused at the testicular level during acute and chronic besnoitiosis. Moreover, biomarkers with prognostic value at the reproductive level could be helpful to discern sterile, subfertile and fertile bulls, since ultrasound analysis is presently the only tool available to determine the extent of genital lesions. However, mild testicular fibrosis is a common genital lesion not necessarily associated with altered semen quality [10]. In this context, the usefulness of acute-phase response biomarkers should be clarified, taking into account their diagnostic value in numerous inflammatory diseases in cattle [25].

We have reported relevant differences among different naturally infected bull groups. These animals were grouped following a restrictive criterion based on a combination of clinical, histopathological, molecular and serological findings to compare homogeneous groups according to the different clinical phases of *B. besnoiti* infection. However, in the present study, sample limitation was unavoidable, since the time post-infection in field animals was unknown. In fact, the tissue cyst size variability observed in some animals evidenced asynchrony in the chronobiology of the infection, as previously noted for acutely infected bulls [6]. Another limitation was the small sample size (only three acutely infected bulls with testicular degeneration were evaluated), which may have also contributed to the fact that no markers of sterility were identified. Nevertheless, this study offers robust data and complements a previous study that identified a few molecular markers related to disease progression in testicular tissues [13]. Moreover, the results obtained in experimentally infected cattle offer valuable data about the kinetics of the acute-phase response during acute and chronic infection, as discussed below.

Haptoglobin, albumin, PON-1 and ADA were identified as the most promising biomarkers for the acute phase of the infection. Haptoglobin was increased, whereas albumin and PON-1 showed a decrease in experimentally infected cattle, and peaks were detected during the first month post-infection. In naturally infected cattle, the lowest values of albumin and PON-1 and the highest values of ADA corresponded to bulls with acute infection.

The Hp biomarker in experimentally infected cattle appeared to be a good predictor biomarker of the infection, as the first peak of Hp levels at 10 dpi appeared simultaneously in all infected groups and coincided with the incubation period and enlarged lymph nodules just prior to fever development (between 7 and 24 dpi) [17], except for the subcutaneously inoculated group, which presented fever at 7 dpi. This peak was also detected prior to innate immune response interferon (IFN) levels (12–22 dpi) and seroconversion (19–25 dpi). A second increase in Hp levels was detected at approximately 22 and 35 dpi in the intravenously and intradermally inoculated groups, respectively, which may be related to a delay in seroconversion in both groups and the higher severity of clinical signs observed in the intradermally infected group. According to the concentration of Hp, both the subcutaneously and intradermally infected groups presented higher Hp concentrations of 1–2 g/l, while Hp levels were lower in the intravenously infected group (between 0.2–1 g/l), probably related to the severity of the infection, as stated by others [25, 26]. In fact, there are several examples of the diagnostic and prognostic value of serum Hp in mastitis, enteritis, peritonitis, pneumonia, endocarditis and endometritis in cattle [25, 26]. However, Hp values did not significantly vary in naturally infected animals despite the severity of the infection, particularly in bulls with early acute infection [6]. In our study, Hp levels were in the range of 0.1–0.15 g/l in all naturally infected bulls, similar to the low Hp serum concentrations (< 20 mg/l) reported in healthy cattle [25]. These results might be explained by the narrow diagnostic window of Hp levels observed in experimentally infected cattle, where the first peak was observed prior to fever development. Bulls with early acute infection had already developed fever a few days prior to sampling. Another feasible explanation could be that the concentration of Hp may decrease dramatically during accelerated haemolysis [27]. However, our serum samples were not apparently haemolysed.

Albumin and PON-1 peak detection varied among groups at 15 dpi in the subcutaneously inoculated group and 25 dpi in the intradermally and intravenously inoculated groups around humoral (seroconversion; 19–25 dpi) and cellular adaptive immune responses (adaptive immune response IFN levels; 12–19 dpi). A significant decrease in albumin concentrations was also observed in acutely infected bulls that agrees with the decrease in serum albumin levels also reported in naturally infected bulls with acute and subacute *B. besnoiti* infection by Langenmayer et al. [23]. These findings indicate that the concentrations of albumin may reflect the magnitude of changes and predict adverse clinical outcomes. Vincent et al. [28] suggested that low concentrations of albumin in the blood serum serve as an important prognostic indicator that is associated with increased mortality and morbidity. In fact, three naturally infected bulls died during the acute phase with respiratory clinical signs, fever and orchitis [6]. On the other hand, it is possible that the reduction in PON-1 levels in acutely infected animals coincides with endothelial dysfunction, as stated by others [29]. Indeed, severe vascular lesions were found in the pampiniform plexus of acutely infected bulls [6].

ADA is an important enzyme that participates in neuromodulation, apoptosis, necrosis and proliferation of lymphocytes during cellular response [30]. According to Franco et al. [31], ADA acts during inflammation in injured tissue, i.e., regulates the concentration of extracellular adenosine, an important molecule with anti-inflammatory properties, since this enzyme converts adenosine to inosine.

In our study, ADA levels from acutely infected bulls were higher than in the other groups of bulls (significant differences with non-infected and subclinically infected bulls). This increase in ADA activity has been described in the course of diseases that induce a cellular immune response, as for example in neosporosis [32] and toxoplasmosis [33]. Thus, increased ADA activity leads to decreased adenosine levels, which might regulate to the inflammatory process and tissue damage induced by *B. besnoiti* infection.

In general terms, apart from a suggested correlation of APP levels with acute besnoitiosis severity, this acute response appears to be related to a few upregulated molecular markers (IL-6, IL-1α, PLAT and TIMP1) detected in the testicular parenchyma of acutely infected bulls versus a downregulation of PLAT, IL-1α, IL-6, IL-8 and IL-10 in chronically and subclinically infected bulls [13]. APPs are predominantly glycoproteins synthesised by hepatocytes in response to IL-6, tumour necrosis factor alpha (TNF-α), and other proinflammatory cytokines such as IL-1α [34]. This relationship between overexpression of pro-inflammatory cytokines and high levels of APPs has been described in experimental *Trypanosoma evansi* infections in rats [35].

High total protein and globulin values were characteristic of chronically infected cattle, in agreement with the observations made by Langenmayer et al. [23, 36], who reported high levels of serum total proteins and globulins in cows with chronic *B. besnoiti* infection. In addition, higher antibody levels have been associated with severely chronically affected animals [37]. Moreover, high values of globulins have been described in cows with chronic diseases [38], high values of total proteins in cows diagnosed with endometritis [39] and chronic lameness [40]. Globulin concentrations above 50 g/l are an indicator of chronic inflammation [41]. In our study, animals with chronic infection, in addition to having values of 70 g/l (Fig. 2), also presented an intense inflammatory infiltrate with the presence of lymphocytes, some macrophages, abundant young granulation tissue and an abundance of fibroblasts in the testicular parenchyma [13].

Markers of sterility were not identified, since acutely and chronically infected sterile bulls did not show a similar pattern of acute-phase response compared to non-infected and subclinically infected animals. The most relevant finding was that AChE values were higher in late acutely infected bulls, and significantly higher levels of AChE were detected in chronically infected animals than in subclinically infected animals. An increase in AChE activity may lead to enhanced degradation of acetylcholine, a molecule that has an anti-inflammatory effect [42]. Therefore, the presence of an intense inflammatory infiltrate observed in natural infection [6] in both acute and chronic besnoitiosis may be associated with an increase in AChE activity. Remarkably, AChE values were higher in calves (≈ 0.5 μmol/ml·min) than in bulls (≈ 0.3 μmol/ml·min), even though both groups were classified as subclinically infected cattle. A more severe infection in experimentally infected cattle could explain this finding, since microscopic lesions (e.g., thrombus, oedema, inflammation, hyperkeratosis and dilated sweat glands) were detected in testes from infected calves [43].

The levels of the different biomarkers studied in experimentally infected calves at 0 dpi and in non-infected field cattle were very similar except for PON-1, which was lower in field animals than in experimentally infected calves. Thus, PON-1 levels could be influenced by animal age. It has already been described that elderly people have lower PON-1 levels than adults and children [44].

## Conclusions

We have described the pattern of the acute-phase response during acute and chronic bovine besnoitiosis. The results have identified several biomarkers with promising diagnostic value. The biomarkers associated with an acute infection (Hp, albumin, PON-1 and ADA) are related to previously reported molecular biomarkers in the testicular parenchyma of infected bulls and could help in the diagnosis of early infections and complement IgM detection.

On the other hand, the value of AChE associated with elevated globulin and total protein levels as indicative of testicular injury remains to be elucidated. The kinetics of the different biomarkers should be corroborated in future longitudinal field studies, since biomarkers could aid in decisions regarding the use of seropositive bulls for natural mating.

## Data Availability

Data supporting the conclusions of this article are included within the article.

## Abbreviations

AChE: Acetylcholinesterase
ADA: Adenosine deaminase
APPs: Acute-phase proteins
Hp: Haptoglobin
ICAM: Intercellular adhesion molecule
IFN: Interferon
IL-1α: Interleukin-1 alpha
IL-6: Interleukin-6
IL-8: Interleukin-8
IL-10: Interleukin-10
MMP13: Metalloproteinase 13
PLAT: Tissue-type plasminogen activator
PON-1: Paraoxonase-1
TIMP1: Matrix metalloproteinase inhibitor-1
TNF: Tumour necrosis factor
